# Multisystemic Disease in a Child and Successful Recovery With Antiviral Treatment

**DOI:** 10.7759/cureus.53893

**Published:** 2024-02-09

**Authors:** Ana Sofia Rodrigues, Aida Correia de Azevedo, Susana Nobre, Paula Fonseca

**Affiliations:** 1 Pediatrics, Centro Hospitalar do Médio Ave, Vila Nova de Famalicão, PRT; 2 Paediatric Hepatology and Liver Transplantation Unit, Centro Hospitalar e Universitário de Coimbra, Coimbra, PRT

**Keywords:** immunocompetent, hepatosplenomegaly, cmv infection, cholestatic hepatitis, acute liver failure

## Abstract

Cytomegalovirus (CMV), a member of the *Herpesviridae* family, typically causes asymptomatic infections or mild mononucleosis-like syndromes in immunocompetent individuals. However, severe manifestations are well-documented in immunocompromised populations.

This case report presents a previously healthy seven-year-old girl with a rare and complex presentation of primary CMV infection leading to severe multiorgan involvement, hepatosplenomegaly, cholestasis, bicytopenia, and a prolonged disease course. The patient's condition prompted an exhaustive diagnostic investigation, ruling out other potential causes. The diagnosis was confirmed by positive CMV IgM and IgG antibodies and a significantly elevated CMV DNA viral load. Treatment with intravenous ganciclovir resulted in a remarkable recovery.

The case underscores the importance of considering CMV as a potential etiology of hepatitis, even in immunocompetent children, and the challenges of diagnosing complicated CMV infections. While guidelines for treating CMV in immunocompetent individuals are lacking, this report suggests that antiviral therapy may be beneficial in severe cases. Further research is needed to establish clear treatment protocols for such instances. This report contributes to the limited body of literature on severe CMV-induced hepatitis in immunocompetent children, emphasizing the need for heightened clinical awareness and timely interventions to prevent progression to acute liver failure.

## Introduction

Cytomegalovirus (CMV), also known as human herpes virus 5 (HHV 5), is a double-stranded DNA virus member of the *Herpesviridae *family [1, 2]. It is involved in a wide spectrum of diseases in humans and is an endemic virus in most areas of the world [3]. It presents a variable geographical seroprevalence, ranging from 40% to 100% [4-5].

Previously healthy children with an acquired CMV infection are usually asymptomatic. In 10% of the cases, it may present as a mononucleosis-like syndrome [6], characterized by the presence of fever, fatigue, pharyngitis, adenopathy, and hepatitis, which is usually benign and self-limited [5-8]. More severe presentations, including fulminant hepatic failure [1, 9], pneumonitis, encephalitis, thrombocytopenia, vasculitis, colitis, and retinitis, are well described in cases of immunocompromised children, congenital or perinatal CMV infections, or acquired infections in premature infants. However, these complications are thought to be rare in immunocompetent children. Misdiagnosis and a lack of reporting may also play a role in this apparently low prevalence [1,2,3,5-8]. 

The authors present a case of a primary CMV infection in a previously healthy girl who developed a severe and prolonged disease course with multiorgan system compromise and for whom antiviral treatment was used.

## Case presentation

A previously healthy seven-year-old girl was observed on the first day of fever in the emergency department of a level 2 hospital in north Portugal. She was referring to chest pain, coughing, and odynophagia. The chest X-ray was normal, and rapid oropharyngeal antigen testing for group A streptococcal infection was negative. However, as the clinical suspicion was high, she was treated with amoxicillin (50 mg/kg/day, twice a day) for five days for possible bacterial tonsillitis.

On day seven of the disease, she returned to our hospital with an intermittent high fever (axillary temperatures ranged from 38.5°C to 40°C) and worsening of the general condition within 24 hours of evolution, now presenting emesis, abdominal pain, distension, and prostration. She denied other symptoms like rhinorrhea, nausea, diarrhea, muscle aches, and joint pain.

It was mentioned that there was previous contact with a dog tick a few weeks before the beginning of the symptoms. There wasn’t any illness among the cohabitants, and she denied traveling abroad.

**Figure 1:**
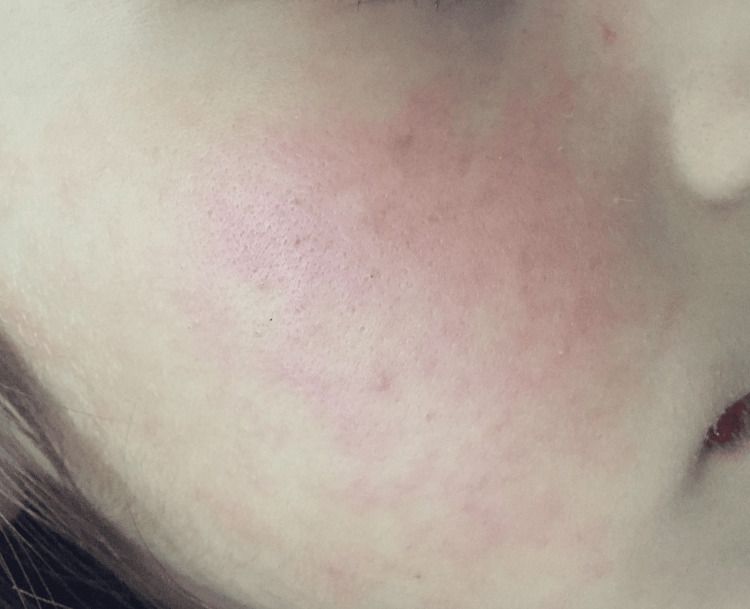
A macular rash was seen on the patient.

On admission, she was of ill appearance; her axillary temperature was 38.5ºC and she had tachycardia, tachypnea, and normal blood pressure. She presented with mucosal dryness, jaundice, and an erythematous, non-pruritic macular rash (Figure 1).

**Figure 2:**
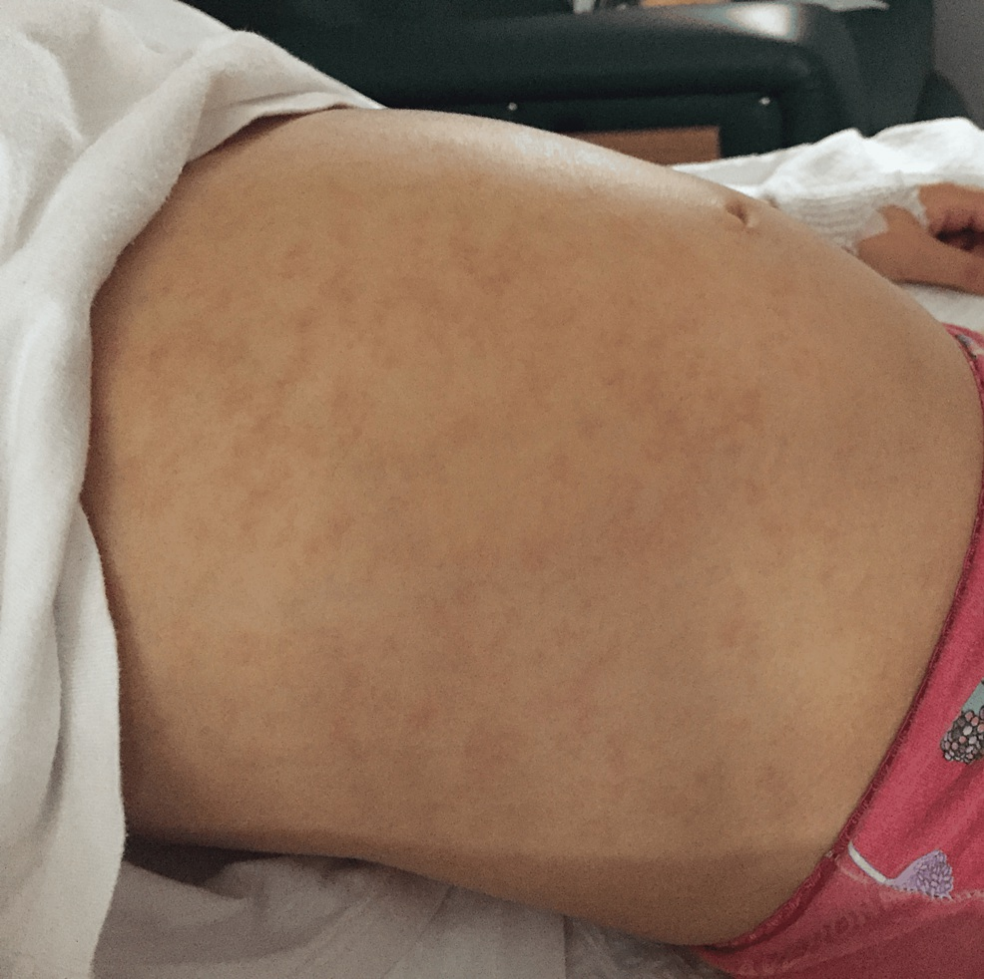
Abdominal distension was observed in the patient.

Bilateral retroauricular lymph nodes were palpable, small, mobile, soft, non-tender, not painful, and without change in the underlying skin. The abdomen was distended with diffuse abdominal pain, and the liver and spleen were palpable 3 cm below the costal margin (Figure 2).

A complete blood count revealed a hemoglobin of 10.8 g/dL (normal range (NR): 11.5-15.5 g/dL) and a platelet count of 144 × 10^3 /uL (NR: 150-450×^3/uL). The total white blood cell (WBC) count was 13.26 × 10^3 /uL (NR: 5.0-14.5x10^3/uL), with 49% neutrophils (NR: 33%-76%), 40.9% lymphocytes (NR: 15%-61%), and 7.8% monocytes (NR 0%-5%). Urine and blood cultures were performed and were negative for infections.

The biochemical profile showed cytolysis and cholestasis (Table 1).

**Table 1:** Progression of hemoglobin levels, platelet count, WBC count, hepatic cytolysis and cholestasis enzymes, albumin, total protein, INR, PT, PTT, fibrinogen, D-dimer, CRP, and CMV DNA viral load from the onset of the disease until its resolution. In the table, increased values are marked with “H” and decreased values with "L". WBC: white blood cell; TGO: glutamic-oxalacetic transaminase (aspartate aminotransferase); TGP: glutamic pyruvic transaminase (alanine aminotransferase); LDH: lactate dehydrogenase; ALP: alkaline phosphatase; GGT: gamma-glutamyl transpeptidase; TB: total bilirubin; DB: direct bilirubin; INR: international normalized ratio; PT: prothrombin time; PTT: partial thromboplastin time; CRP: C-reactive protein (CRP); CMV: cytomegalovirus

	Reference values for the sex and age of the patient	Days of disease					Hepatology consultation	
		Admission (Day 7)	Day 8	Day 9	Day 10	Day 13	1 month	2 months
Hemoglobin (g/dL)	11.5-15.5	10.8 (L)	10.2 (L)	10 (L)	10.22 (L)	10.6 (L)	11.23 (L)	13.3
Platelet count (x10^3/uL)	150-450	144 (L)	65 (L)	36 (L)	27 (L)	199	274	296
WBC (x10^3/uL)	5.0-14.5	13.2	15.9 (H)	25 (H)	23.7 (H)	8.45	8.45	6.45
Neutrophils (%)	33-76	49	23 (L)	15 (L)	14 (L)	35	48	42
Lymphocytes (%)	15-61	41	58	72 (H)	86 (H)	40	31	23
Monocytes (%)	0-5	7.8 (H)	17.4 (H)	9.9 (H)	10.2 (H)	4	2	5.3
Hepatic cytolysis								
TGO (U/L)	15-40	177 (H)	180 (H)	133 (H)	105 (H)	87 (H)	38	35
TGP (U/L)	10--35	166 (H)	129 (H)	118 (H)	96 (H)	92 (H)	30	26
LDH (U/L)	110-295	498 (H)	444 (H)	547 (H)	441(H)	456 (H)	-	-
Cholestasis enzymes								
ALP (U/L)	175-420	334	-	381	426 (H)	630 (H)	190	175
GGT (U/L)	13-25	105 (H)	-	188 (H)	239 (H)	424 (H)	57 (H)	12
TB (mg/dL)	0.2-1.0	7.9 (H)	7.24 (H)	7.6 (H)	8.1 (H)	8.6 (H)	1.5 (H)	0.7
DB (mg/dL)	<0.35	5.37 (H)	5.15 (H)	6.1 (H)	6.4 (H)	2.9 (H)	1 (H)	-
Albumin (g/dL)	3.7-5.5	-	2 (L)	2.4 (L)	3.3 (L)	3.5 (L)	4	4.5
Total protein (g/dL)	6-8	-	4.2 (L)	4.6 (L)	4.9 (L)	5.6 (L)	6.3	6.6
Coagulation								
INR	0.8-1.2	1.35 (H)	1.64 (H)	1.8 (H)	1.27 (H)	1.13	1.11	1.17
PT (sec)	12.2-15.5	14.7	19 (H)	19 (H)	-	-	13	14
PTT (sec)	26.5-35.5	29.6	33.6	30	-	-	29	32
Fibrinogen (mg/dL)	220-440	268	139 (L)	91 (L)	83 (L)	127 (L)	-	-
D-Dimer (ng/mL)	≤ 400	5241 (H)	4812 (H)	-	4279 (H)	4146 (H)	352	-
CRP (mg/dL)	< 1	24.3	8.1	6.2	3.9	2.4	<0.1	<0.1
CMV DNA viral load (copies/ml)	Undetectable	-	-	-	-	7996 (H)	498 (H)	Undetectable

The C-reactive protein (CRP) level was 24.30 mg/dL (NR: <1 mg/dL), and renal function was normal. Coagulation tests were abnormal; the international normalized ratio (INR) was 1.64 (NR: 0.8-1.2), fibrinogen was 139 mg/dL (NR: 220-440 mg/dL), and D-dimer levels were 4,812 ng/mL (NR: ≤400 ng/mL), with normal prothrombin time (PT) and normal activated partial thromboplastin time (APTT). She also presented with proteinuria and bilirubinuria. Abdominal ultrasound (US) revealed hepatosplenomegaly (larger diameters of 17.6 cm and 13.6 cm, respectively) and small-volume pelvic ascites. A second chest X-ray was performed, revealing a right pleural effusion.

An extended screening for infectious agents was performed, revealing positive IgM and IgG anti-CMV antibodies (38.09 UA/mL (positive ≥6) and 44.9 UUA/mL (positive ≥1), respectively. Tests for IgM and IgG Epstein-Barr virus (EBV) and nuclear antigen antibody (EBNA) were also positive (past infection). Complement levels and immunoglobulin levels were normal.

Further investigation for *Leptospira*, *Rickettsia*, *Leishmania*, hepatitis A virus antibodies, hepatitis B surface antigen, hepatitis C virus antibodies, hepatitis D virus antibodies, hepatitis E virus antibodies, human immunodeficiency virus antibodies, and herpes simplex virus antibodies was required. Blood cultures and CMV polymerase chain reaction (PCR) tests were also requested.

The child was admitted to the pediatric department of the hospital in north Portugal. Considering the hypothesis of sepsis with multiorgan failure or rickettsiosis fever, empirical antibiotherapy was initiated with cefotaxime (120 mg/kg/day) and azithromycin (10 mg/kg/day).

After 24 hours (day eight), the blood profile showed worsening (Table 1) of bicytopenia (anemia and thrombocytopenia), and the peripheral blood film showed atypical lymphocytes. It also revealed hypoproteinemia with hypoalbuminemia, hypokalaemia (potassium level: 3.2 mEq/L, NR: 3.5-5.5 mEq/L), elevated ferritin (334 ug/L, NR: 36-92 ug/L), elevated triglycerides (301 mg/dL, NR: 20-10 mg/dL), and low total cholesterol (73 mg/dl, NR: 135-299).

Due to clinical and analytical deterioration and the risk of progression to acute liver failure, the patient was transferred on day nine to the pediatric hepatology and liver transplantation unit of a hospital in central Portugal.

On the next day (day 10), she exhibited signs of clinical improvement, including apyrexia for over 24 hours. Physical examination showed improvement of general appearance with an evanescent macular rash and a less distended and painful abdomen (abdominal perimeter was 58 cm), and there was a weight loss of 1 kg. Despite clinical improvement, there was an analytical worsening. The leukogram showed marked lymphocytic pleomorphism, suggestive of a viral infection (Table 1). Treatment with ursodeoxycholic acid (UDCA) at 15 mg/kg/day, once a day, was initiated.

The immunology evaluation, consisting of lymphocyte subset studies, serum immunoglobulin and IgG subclass levels, and pneumococcal antibody titers, was normal. Serologies for *Leptospira*, *Rickettsia*, *Leishmania*, hepatitis A virus antibodies, hepatitis B surface antigen, hepatitis C virus antibodies, hepatitis D virus antibodies, hepatitis E virus antibodies, human immunodeficiency virus antibodies, and herpes simplex virus antibodies were negative.

After six days of hospitalization (day 13), a PCR test for CMV came up positive, showing a value of 7,996 copies/mL, indicating a disseminated CMV infection. Intravenous ganciclovir 5 mg/kg every 12 hours was initiated, and antibiotic therapy was suspended (after completing three days of azithromycin and seven days of cefuroxime).

The patient kept recovering both clinically and analytically and was discharged on the 17th day of illness after completing a total of seven days of UDCA and four days of intravenous ganciclovir.

She maintained follow-up with the hepatology department and continued treatment with oral valganciclovir and UDCA (completed a total of a 10-week course of valganciclovir (30 mg/kg/day, twice daily) and a six-week course of UDCA (15 mg/kg/day, once daily)). A plasma CMV PCR test was repeated 10 days later, showing a decrease in the viral load (498 IU/mL).

In a follow-up evaluation two months later, it was seen that the patient had a complete recovery, with normalization of all analytical parameters. The CMV DNA load was undetectable, and the US showed normal liver and spleen size without peritoneal effusion.

Table 1 shows the progression of hemoglobin levels, platelet count, white blood cell count, hepatic cytolysis and cholestasis enzymes, INR, PT, APTT, CMV DNA viral load, and CMV IgM from the onset of the disease until its resolution.

Given that all other causes of hepatitis were excluded, it was safely concluded that the patient had acute CMV hepatitis. The diagnosis of primary CMV infection was confirmed by a positive IgM CMV, a positive IgG CMV, and a highly positive PCR for CMV.

## Discussion

Mononucleosis-like syndrome, mostly caused by the EBV virus, was initially described as glandular fever in 1889 and later, in 1920, was used to describe a febrile illness characterized by absolute lymphocytes and atypical mononuclear cells in the blood. In 10% of cases, infectious mononucleosis (IM) is caused by other agents like CMV, HIV, toxoplasma, human herpesvirus-6 (HHV-6), and hepatitis B [10]. In the case of CMV, infection occurs most often when the immune system is compromised. In immunocompetent children, the infection can present as a mononucleosis-like syndrome [4,10] which is self-limited, and the majority of children experience resolution of symptoms over several days or weeks without sequelae [4].

Our case was particular since many rare complications occurred in a single child. In fact, we were in the presence of a previously healthy girl with fever, macular rash, ascites, hepatosplenomegaly, atypical leukocytes, highly elevated liver enzymes, coagulopathy, parameters of cholestasis, and bicytopenia. During the clinical course, an exhaustive investigation of possible treatable causes of cholestasis was performed, and several diagnostic hypotheses were raised, which were CMV or EBV infection, viral hepatitis, sepsis, rickettsial infection, leptospiral infection, bacterial infection by subtherapeutic antibiotic dosing, and hemophagocytic lymphohistiocytosis syndrome. Acute EBV infection was excluded because EBNA was positive, so it was compatible with a previous infection (two to four months before).

A CMV infection diagnosis is based on specific anti-CMV serum IgM positivity or the increase of specific anti-CMV IgG titers by more than four times the normal range. Detection of blood CMV DNA via PCR is also useful to establish the diagnosis [11,12]. In our case, the diagnosis was made taking into account the clinical course and serological tests (positive CMV IgM and CMV IgG antibodies) and confirmed by the quantification of CMV DNA by real-time PCR.

According to the literature, the liver is preferentially affected in 70%-90% of cases, with a mixed hepatocellular and cholestatic pattern [6, 13]. Transaminase elevation is the most common subclinical finding in these patients. Cholestatic hepatitis is a relatively common feature of CMV hepatitis occurring during early childhood and has been associated with more severe disease [8, 11]. However, in a study with 49 immunocompetent pediatric patients in which all of them had cholestatic hepatitis, they all recovered completely without any chronicity [11]. Rash and hepatomegaly are present in 33% and 10%-38% of patients, respectively [14]. Atypical leukocytes are almost always seen [7]. Hematologic abnormalities, such as thrombocytopenia, pancytopenia, disseminated intravascular coagulation, and myelodysplastic changes, can also be present. However, only a few cases have been reported in the pediatric age group [5].

Previous cases of acute fulminant hepatic failure due to CMV infection, requiring an emergency liver transplant, have already been described in the literature [9, 15, 16]. In this case, although INR was responsive to vitamin K administration, the clinical and analytical deterioration led us to consider this possible outcome.

Therefore, timely diagnosis and treatment prevent the progression of acute liver failure. The prevalence of acute liver failure is unknown in pediatric patients [17]. It is one of the most challenging medical emergencies due to its prognostic and therapeutic implications [17].

Extensive investigation and rapid identification of etiologic agents are extremely important in cases of acute liver dysfunction [17]. Acute hepatitis in children can be caused by a large number of agents, such as primary hepatotropic (hepatitis A-E) and non-hepatotropic viruses such as CMV, EBV, herpes simplex virus, enterovirus, adenovirus, HHV-6, and rubella. The latter accounts for up to 10% of all viral hepatitis and may cause severe liver disease, especially in neonates and immunocompromised patients [14]. However, the percentage of cases in which progression to acute liver dysfunction occurs remains unknown in immunocompetent children. Previous reports indicate that CMV, EBV, and HHV-6 have been associated with the prognosis of organ transplantation [18, 19]. Other acute liver failure etiologies should also be considered, including drug exposure, metabolic disorders, and autoimmune diseases [18-20].

Reports of severe clinical manifestations of CMV infection in immunocompetent children are limited [9]. Currently, no clear guidelines are available for the treatment of CMV hepatitis in immunocompetent hosts, and treatment with antiviral agents is still controversial [1,4,11,15]. For immunocompetent individuals, treatment is usually not required [1,2,6-9,11,13,14,17]. The severity and potential morbidity of CMV disease must be balanced against the potential side effects of the treatment, including myelosuppression (neutropenia), central nervous system disorders, hepatotoxicity, irreversible infertility (inhibition of spermatogenesis), or teratogenesis [6]. 

However, effective treatment with ganciclovir has been reported in cases of severe and persistent CMV mononucleosis-like syndrome that prove to be unresponsive to supportive therapy. Antiviral treatment is associated with a faster recovery [11,13] and is known to be effective in preventing CMV-induced acute liver failure [13]. According to the literature, CMV liver infection without involvement of other organs in children does not require antiviral treatment and usually has a great outcome [11,13].

Therefore, treatment decisions should be based on a combination of factors that assess host immune status, viral load, and severe acute CMV infection [1,6]. When treatment is needed, it should be given for a minimum of two to three weeks. Treatment should continue until there is documented clearance of the virus from the blood [6]. 

Antiviral therapy was also suggested to be effective and safe for immunocompetent children with CMV infection with associated thrombocytopenia [20]. A recent study reports that around 35% of children require antiviral therapy for disease control. Antiviral agents may be considered if thrombocytopenia is refractory to intravenous immunoglobulin (IVIG) therapy or if concomitant hepatitis, gastritis, pneumonitis, or Evans syndrome are present [20]. 

In our case, UDCA treatment was initiated, taking into account our child's cholestatic hepatitis. The decision to initiate IV ganciclovir for four days followed by oral valganciclovir was made based on the high CMV viral load and severe, persistent, and progressive clinical course. It must be mentioned that without the serology for CMV and CMV viral load, it would have been impossible to determine the cause, confirming the importance of these tests. Antiviral treatment resulted in a complete recovery.

Therefore, despite limited evidence, in complicated cases and patients with multiorgan involvement, antiviral treatment should be considered [1,2,6,8,9,11,13,14,17]. However, further research is needed to establish treatment guidelines.

## Conclusions

In conclusion, we consider that our case highlights multiple and uncommon complications of a CMV infection in a previously healthy child. To our knowledge, this is one of the few cases reported of CMV-induced hepatitis with severe, prolonged, and multisystemic disease in an immunocompetent child, requiring hospitalization, transfer to a pediatric liver transplantation unit, and treatment with an antiviral agent. This case also emphasizes the importance of investigating CMV as a hepatitis etiologic agent, independent of the patient's immune status, and that antiviral treatment should be considered in severe cases.
